# Dual-functionality of *Thauera* sp. JM12B12: aerobic denitrification and bioflocculation for nitrogen and suspended particles removal at low carbon-to-nitrogen ratios

**DOI:** 10.3389/fmicb.2025.1730924

**Published:** 2025-12-18

**Authors:** Mingxia Zhang, Yulian Zhang, Qing Yao, Yanna Hu, Honghui Zhu

**Affiliations:** 1 Key Laboratory of Agricultural Microbiomics and Precision Application (MARA), Key Laboratory of Agricultural Microbiome (MARA), Guangdong Microbial Culture Collection Center (GDMCC), Guangdong Provincial Key Laboratory of Microbial Culture Collection and Application, State Key Laboratory of Applied Microbiology Southern China, Institute of Microbiology, Guangdong Academy of Sciences, Guangzhou, China; 2 Key Laboratory of Biology and Genetic Improvement of Horticultural Crops (South China), Ministry of Agriculture and Rural Affairs, Guangdong Province Key Laboratory of Microbial Signals and Disease Control, Guangdong Engineering Research Center for Litchi, College of Horticulture, South China Agricultural University, Guangzhou, China

**Keywords:** aerobic denitrification, flocculation activity, genome analysis, low C/N ratio, *Thauera*

## Abstract

Denitrifying bacteria with flocculation capacity were dual-function microorganisms that can simultaneously remove nitrogen (N) and reduce suspended particles in wastewater, providing a sustainable bioremediation strategy. In this study, a novel denitrifying bacterium capable of producing bioflocculants, *Thauera* sp. JM12B12, was isolated and investigated. The results confirmed that this strain could completely remove NO_3_^−^-N and NO_2_^−^-N under microaerobic conditions with a low C/N ratio of 5, using lactate as the optimal carbon source. Notably, no other harmful inorganic N species were produced during denitrification, and total N removal efficiency consistently exceeded 93.0%. Optimal denitrification conditions include a pH range of 7–9, salinity of 0–1.5%, temperature of 25–40 °C, and static incubation. Remarkably, this strain synthesized extracellular bioflocculants during NO_3_^−^-N removal, achieving 91.4% flocculation efficiency with cell-free supernatant. Genome analyses revealed a complete denitrification pathway (possessing *napA*, two *nirS*, *norB*, *nosZ*) and 80 bioflocculant-related genes (polysaccharide production and protein secretion), highlighting its dual capacity for N and suspended particle removal. PCR also confirmed key denitrification genes. Therefore, JM12B12 could be a multifunctional microbial agent for N removal and flocculation, offering a sustainable solution for low C/N wastewater treatment, particularly valuable in recirculating aquaculture systems.

## 1 Introduction

Recirculating aquaculture system (RAS) represented a promising direction for future aquaculture development, offering its energy conservation, water preservation, land efficiency, and no seasonal constraints (Wang F. et al., 2025). With the long-term operation of RASs, the concentrations of nitrate (NO_3_^−^-N) in aquaculture water could increase to 100 mg/L or more, and the high concentration of NO_3_^−^-N threatened aquatic animals and aquatic ecosystems (Li et al., 2023; Ma et al., 2024). Suspended particles (mainly from residual feed and animal feces) were also a major pollutant in aquaculture water, which directly heightened water turbidity and influenced the survival of aquatic animals (Zhang K. et al., 2025). For instance, an overabundance of suspended particles in aquatic systems could induce mortality events in juvenile chum salmon, primarily attributed to respiratory failure caused by gill clogging from particles (Kishi et al., 2025). Therefore, removal of NO_3_^−^-N and suspended particles was of great significance for enhancing the quality of aquatic products.

Biological nitrogen (N) removal has emerged as a cost-effective and environmentally friendly approach to treating N pollution, and traditional N removal relies on nitrifying bacteria and denitrifying bacteria, which require strictly aerobic and anoxic conditions, respectively (Gu et al., 2022). However, to ensure the health and growth of reared animals, the aquatic water usually needs to be maintained in aerobic conditions, which is detrimental to the traditional denitrifying bacteria (Li et al., 2023). Fortunately, the aerobic denitrifying bacteria, which defied traditional metabolic paradigms by reducing NO_3_^−^-N or nitrite (NO_2_^−^-N) to N_2_ under aerobic conditions, have been uncovered. These functional bacteria, such as *Paracoccus*, *Pseudomonas*, *Acinetobacter*, and *Alcaligenes*, have been isolated from a variety of natural environments (Chen Z. et al., 2024; Lan et al., 2023; Li et al., 2025; Wang H. et al., 2025). Moreover, aquatic water has a kind of relatively low carbon-to-N (C/N) ratio, resulting in many aerobic denitrifying bacteria that hardly adapt to the aquatic water environment (Pan et al., 2024). Because of this, the aerobic denitrifying bacteria that achieve efficient denitrification at a low C/N ratio exhibit great application value.

Bioflocculants could reduce the water turbidity by binding suspended particles, and they offered advantages of biodegradability and non-toxicity compared to other flocculants (Show et al., 2024). Bioflocculants were the macromolecular metabolites with flocculation activity produced by microorganisms, primarily consisting of extracellular polymeric substances (EPS), including polysaccharides, proteins, and nucleic acids (Chen W. et al., 2024). Some bioflocculant-producing bacteria have been reported, such as *Bacillus*, *Azoarcus*, and *Pseudomonas* (Alias et al., 2024; Chen L. et al., 2024; Christiaens et al., 2023). Even though several potential bioflocculant-producing strains have already been investigated, studies on denitrifiers possessing this function are still limited at present. Further exploration of bioflocculant-producing denitrifying bacteria was necessary to improve the aquatic water quality.

*Thauera* was the dominant genus in various types of wastewater treatment systems, such as the sequencing batch biofilm reactor, the sequencing batch reactor, and the moving bed biofilm reactor (Yu et al., 2024; Yuan et al., 2023; Zhang et al., 2024). In recent years, several *Thauera* strains have been isolated. For instance, *Thauera* sp. SND5 was capable of removing phosphorus and N through simultaneous nitrification–denitrification and phosphate accumulation (Wang and He, 2020); *Thauera* sp. RT1901 could achieve denitrification and phosphorus accumulation in both microaerobic and anaerobic environments (Ren T. et al., 2024); *Thauera* sp. AutoDN2 could remove nitrate and oxidize sulfide to elemental sulfur under autotrophic conditions (Zhang Y. et al., 2025). Strains of *Thauera* with denitrification capability were commonly reported, however, those simultaneously exhibiting high efficiency denitrification and flocculation functions remain scarce.

In this study, we presented a novel bacterium, *Thauera* sp. JM12B12, isolated from aquaculture water, exhibited the remarkable ability to perform aerobic denitrification, even at a low C/N ratio, and concurrently produced bioflocculants during the process of NO_3_^−^-N removal. The denitrification performance of this strain under various conditions and its flocculation activity during the process of denitrification were investigated. Additionally, JM12B12’s aerobic denitrification pathway and flocculation characteristics were conjectured by analyzing the related functional genes according to the genome annotations. Our findings demonstrated that strain JM12B12 could be a promising candidate for simultaneously removing N and suspended particles from aquaculture water with a low C/N ratio.

## 2 Materials and methods

### 2.1 Medium

For preliminary isolation of the aerobic denitrifying bacteria, a denitrification screening medium with low N (DM-L) was employed. The composition of DM-L medium included the following components per liter: sodium succinate 0.25 g, sodium citrate dihydrate 0.25 g, Na_2_HPO_4_ 1.6 g, KH_2_PO_4_ 1.0 g, NaCl 0.5 g, NaNO_2_ 0.07 g, KNO_3_ 0.1 g, (NH_4_)_2_SO_4_ 0.066 g, MgSO_4_·7H_2_O 0.2 g, 0.2% (v/v) trace element solution (TES), and 0.1% (v/v) mixed carbon source solution (CSS). The pH was adjusted to pH 7.2. Solid plates were prepared by supplementing DM-L with 15.0 g/L agar. Before use, the medium (excluding MgSO_4_·7H_2_O, TES, and CSS) was sterilized by autoclaving at 121 °C (0.11 MPa) for 30 min. Filter-sterilized MgSO_4_·7H_2_O, TES, and CSS were aseptically supplemented to the autoclaved medium. The formulations of TES and CSS followed protocols established in our previous work (Zhang et al., 2022).

### 2.2 Isolation and identification of aerobic denitrifying bacteria

Samples collected from *Penaeus vannamei* aquaculture water in Jiangmen City, Guangdong Province, China, were diluted (10^−1^ to 10^−4^), and 0.1 mL from 10^−2^ to 10^−4^ dilutions was spread on DM-L agar, incubated aerobically at 30 °C for 2–7 days. Colonies with distinct morphology were purified and stored at −80 °C in sterile physiological saline supplemented with 25.0% (v/v) glycerol. For denitrifying bacteria isolation, selected strains were grown statically at 30 °C in DM-L medium with 5.0 mg/L NO_2_^−^-N, and residual NO_2_^−^-N was measured using the Griess reaction.

Strain JM12B12 was cultured on LB agar at 30 °C for 48 h, with colonies observed macroscopically and cells via electron microscopy (H7650, Hitachi). Bacterial genomic DNA was extracted using the HiPure Bacterial DNA Kit (Magen Biotech., China). The 16S rRNA genes were PCR-amplified with primers 27F and 1492R, sequenced by GENEWIZ (Suzhou, China), and aligned against the EzBioCloud database (Chalita et al., 2024). Strain JM12B12 showed the highest NO_2_^−^-N removal efficiency and was further analyzed for morphology, phylogeny, and genome. The 16S rRNA sequences were aligned using MAFFT v7.526 under the L-INS-i iterative refinement (Rozewicki et al., 2019). Maximum-likelihood (ML) phylogenetic tree was reconstructed with IQ-TREE v2.1.2 with integrated ModelFinder for evolutionary model selection, selecting the best model via Bayesian Information Criterion and assessing node support with 1,000 bootstrap replicates (Hoang et al., 2018; Minh et al., 2020). The phylogenetic tree was visualized using MEGA 11 (Tamura et al., 2021).

### 2.3 Nitrogen removal characteristics

Our results showed that JM12B12 exhibited more effective capability in the removal of NO_2_^−^-N compared to other isolates. Therefore, the effects of various conditions on the N removal efficiency of JM12B12 were investigated. Each variable was adjusted independently, and the optimal conditions were applied in subsequent experiments.

A single colony of JM12B12 was grown in LB medium for 12 h, washed, and resuspended to OD_600_ of 1.0. The suspension was inoculated into media to study N removal, testing sodium acetate, citrate, succinate, lactate, glucose, and sucrose as carbon sources at C/N ratios of 1 to 20. The basal medium (BM) without carbon and N sources was formulated as (per liter): Na_2_HPO_4_ 1.6 g, KH_2_PO_4_ 1.0 g, NaCl 0.5 g, MgCl_2_·6H_2_O 0.1 g, D-biotin 5.0 mg, cobalamin 5.0 mg. Furthermore, the effects of various culture conditions on the denitrification of JM12B12 were investigated, including initial pH (5–11), concentrations of NaCl (0–3.0%), temperature (20–45 °C), and shaking speeds (0–200 rpm). The BM was supplemented with NO_3_^−^-N and sodium lactate as sole N and carbon sources, and the C/N ratio was adjusted to 10. After 48 h of incubation, samples were analyzed for OD_600,_ and the relevant culture supernatants were used for the measurement of N concentrations (NO_2_^−^-N, NO_3_^−^-N, and NH_4_^+^-N). Uninoculated media were used as controls.

To evaluate JM12B12’s denitrification process and N balance, sodium lactate-supplemented BM with either NO_2_^−^-N (BM1) or NO_3_^−^-N (BM2) was used. During incubation, samples were taken to measure OD_600,_ and the relevant supernatants were used for detecting concentrations of NO_2_^−^-N, NO_3_^−^-N, and NH_4_^+^-N. Additionally, total N (TN-N) and intracellular N (CN-N) concentrations were measured at 0 h and 48 h. Uninoculated media were used as controls.

### 2.4 Flocculating activity

Strain JM12B12 was cultured in the BM with NO_3_^−^-N and sodium lactate as the sole N and carbon source, respectively. After 48 h of incubation, the bacterial suspension (BS) was centrifuged at 12,000 g for 10 min to obtain cell-free supernatant (CFS) and cells (CE). The flocculation capabilities of BS, CFS, and CE were determined by the kaolin suspension method (Chen L. et al., 2024). Briefly, the experimental flocculation system comprised 2.0 mL sample (cells were resuspended with the physiological saline solution), 3.0 mL 1.0% CaCl_2_ (w/v), and 95.0 mL 4.0 g/L kaolin suspension, with pH maintained at 7.5 via HCl and/or NaOH adjustment. The mixture was stirred at 200 rpm for 2 min, then stirred at 50 rpm for 5 min, and then allowed to settle for 5 min. The 3 mL of the supernatant was aspirated from a consistent depth of 1 cm below the air-liquid interface. The optical density measurement was conducted immediately post-collection using a calibrated spectrophotometer at 550 nm wavelength. The flocculating efficiency (FloE) was calculated according to the equation: FloE (%) = (A-B)/A × 100, where A represented the absorbance values of samples (BS, CFS, and CE) at 550 nm, and B represented the reference absorbance values (sterile culture medium for BS and CFS; physiological saline for CE) at 550 nm.

### 2.5 Complete genome sequencing, annotations, and analysis

The complete genome of strain JM12B12 was sequenced at Shanghai Majorbio Bio-Pharm Technology Co., Ltd. (China) using a hybrid approach combining Nanopore PromethION (Oxford Nanopore, Oxford, UK) and Illumina HiSeq 2,500 (Illumina, Inc., San Diego, CA, USA). For Illumina sequencing, genomic DNA was fragmented to 400–500 bp using Covaris M220, and libraries were prepared with the NEXTFLEX Rapid DNA-Seq kit. For Nanopore sequencing, DNA fragments were repaired, purified, and ligated with sequencing adapters from the SQK-LSK kit before library preparation and sequencing. Raw Illumina reads were filtered using fastp v0.23.0, while Nanopore reads were processed (basecalling, demultiplexing, trimming) with a minimum Q score of 7. Hybrid assembly was performed using Unicycler v0.4.8, and Pilon v1.22 was used for error correction. The complete genome was reconstructed by integrating data from both platforms.

GeneMarkS v4.3, tRNA-scan-SE v2.0.12, and barrnap v0.9 were used to predict coding sequences (CDS), tRNA, and rRNA, respectively. The predicted CDS were annotated using COG (202006) and KEGG (202209) databases via sequence alignment tools. Core genome circular maps, COG, and KEGG analyses were conducted on Majorbio’s Cloud platform (Han et al., 2024). The genome was also annotated with RAST v2.0 under the Classic RAST scheme. Digital DNA–DNA hybridization (dDDH) and average nucleotide identity (ANI) values were calculated using Genome-to-Genome Distance Calculator 3.0 (formula 2) and FastANI (Jain et al., 2018; Meier-Kolthoff et al., 2013). The phylogenomic tree was constructed using Up-to-date Bacterial Core Gene sets (UBCGs) (Riesco and Trujillo, 2024).

### 2.6 Detection of the key genes for denitrification

Genomic DNA of strain JM12B12 was extracted using the method described in Section 2.2. Primer sets napA_5F/ napA_3R, nirS1_5F/ nirS1_3R, nirS2_5F/ nirS2_3R, norB_5F/ norB_3R, and nosZ_5F/ nosZ_3R were utilized to amplify the denitrification genes from JM12B12 (Supplementary Table S2). PCR amplification was performed using 2 × Phanta Max Master Mix (Vazyme Biotech, Nanjing, China) in a total reaction volume of 20 μL, comprising the following components: 10 μL of 2 × Phanta Max Master Mix, 0.4 μL of primers (10 μM each), 1 μL of DNA template (50 ng/μL), and 8.2 μL of ddH_2_O. PCR reaction program was as follows: 95 °C for 5 min; 30 cycles consisting of 95 °C for 30 s, 58 °C for 30 s, and 72 °C for 2 min 30 s; followed by 72 °C for 10 min. The PCR products were verified by comparing with Trans2K® Plus II DNA Marker (TransGen Biotech, Beijing, China) electrophoretically using a 1.5% agarose gel.

### 2.7 Analytical methods

NO_2_^−^-N, NO_3_^−^-N, and NH_4_^+^-N concentrations were quantified using *N*-(1-naphthyl) ethylenediamine dihydrochloride, ultraviolet spectrophotometry, and Nessler’s reagent photometry at the wavelength of 420 nm, respectively (Baird et al., 2017). TN-N and CN-N concentrations were quantified using alkaline potassium persulfate digestion followed by spectrophotometric detection at 220 nm and 275 nm, respectively (Baird et al., 2017). Standard curves for the determination of N concentrations were shown in Supplementary Figure S1. OD_600_ was determined at a wavelength of 600 nm. N removal efficiency was calculated by the following formula: N removal efficiency (%) = ([N_x_]^i^ - [T3N]^f^)/[N_x_]^i^ × 100, where [N_x_]^i^ is the initial NO_3_^−^-N, NO_2_^−^-N, and NH_4_^+^-N concentration, respectively; [T3N]^f^ is the sum of NO_3_^−^-N, NO_2_^−^-N, and NH_4_^+^-N concentrations. All experiments were carried out in quadruplicate, and results were expressed as the mean of four replicates ± standard deviation (mean ± SD).

## 3 Results and discussion

### 3.1 Isolation and identification of JM12B12

**Figure 1:**
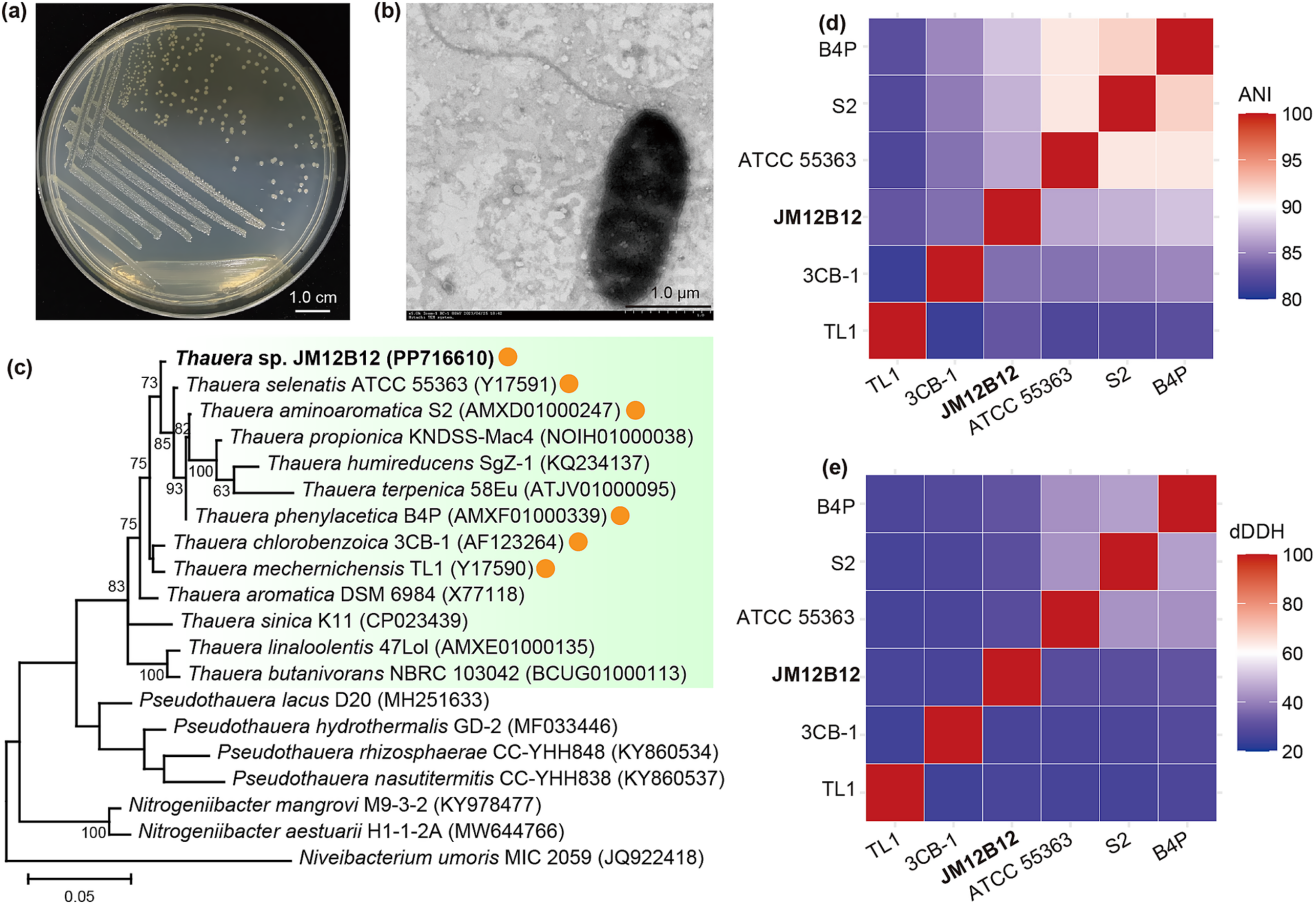
Comprehensive morphological, phylogenetic, and comparative genomic characterization of *Thauera* sp. JM12B12. **(a)** Colonies. **(b)** Cell morphology. **(c)** ML tree from 16S rRNA sequences of JM12B12 and related type strains. **(d,e)** ANI and dDDH similarities between JM12B12 and strains (orange-circled) with high 16S rRNA sequence similarity.

There were 10 isolates obtained, and strain JM12B12 exhibited excellent performance in removing NO_2_^−^-N (Supplementary Table S1). The colonies of strain JM12B12 appeared off-white, opaque, with a smooth surface, regularly circular, and neat-edged, measuring approximately 1 mm in diameter (Figure 1a). Cells of this strain were observed to be rod-shaped (0.7–0.9 × 1.9–2.5 μm) with a single polar flagellum (Figure 1b). The 16S rRNA gene sequence of JM12B12 obtained through PCR amplification (1,400 bp) showed complete identity with the corresponding sequences extracted from its genomic DNA (1,537 bp). The complete 16S rRNA gene sequence was deposited in the NCBI GenBank database under accession number PP716610. The sequence comparison showed that JM12B12 shared the highest similarity with *T. chlorobenzoica* 3CB-1 (99.2%), followed by strains *T. selenatis* ATCC 55363 (99.2%), *T. aminoaromatica* S2 (99.1%), *T. phenylacetica* B4P (99.0%), and *T. mechernichensis* TL1 (98.9%). Phylogenetic reconstruction based on the 16S rRNA sequences revealed that JM12B12 formed a distinct clade within the genus *Thauera* cluster, supported by 73% bootstrap values (Figure 1c). Therefore, JM12B12 was taxonomically assigned to the genus *Thauera*.

### 3.2 Nitrogen removal characteristics of JM12B12

#### 3.2.1 Effect of carbon source and C/N on nitrogen removal efficiency

**Figure 2:**
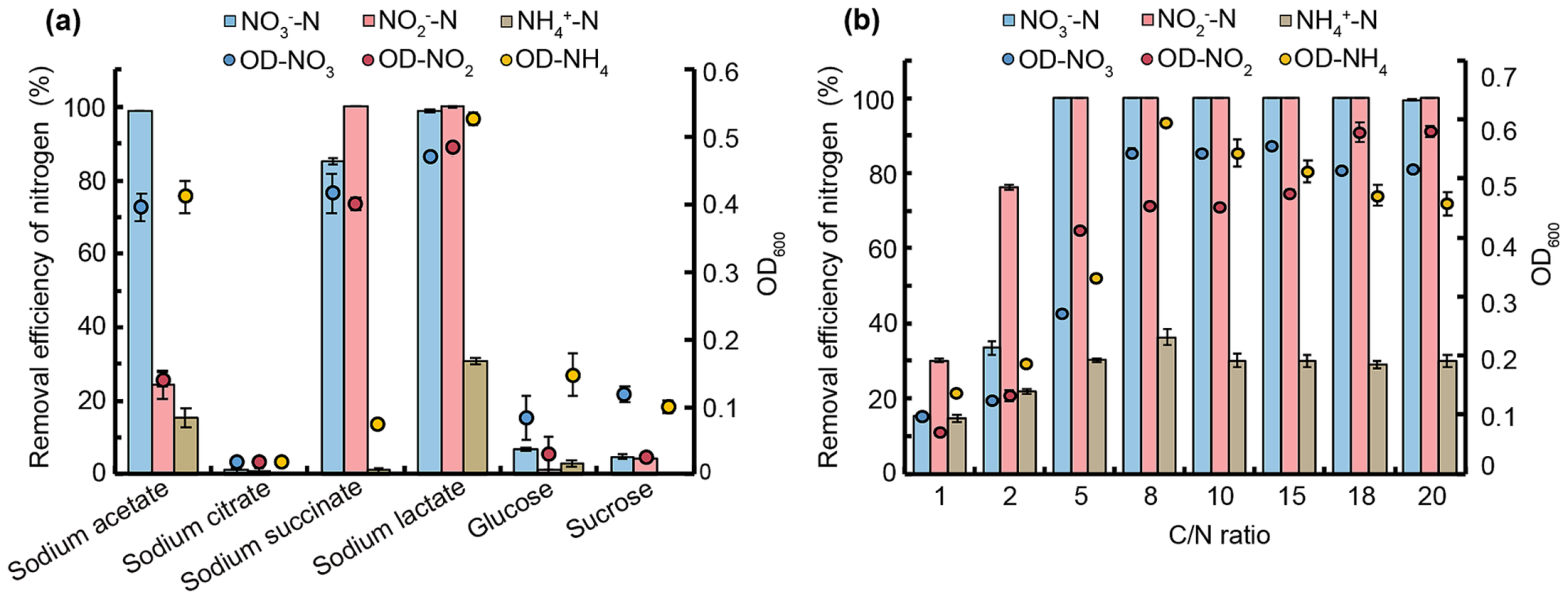
Impact of carbon sources and C/N ratios on NO_3_^−^-N, NO_2_^−^-N, and NH_4_^+^-N removal by strain JM12B12. **(a)** Carbon sources. **(b)** C/N ratios. Data shown as mean ± SD (*n* = 4). OD-NO_3_, OD-NO_2_, and OD-NH_4_ represent OD_600_ values with NO_3_^−^-N, NO_2_^−^-N, and NH_4_^+^-N as sole nitrogen sources, respectively.

To evaluate N removal efficiencies, strain JM12B12 was tested with different carbon sources at a fixed C/N ratio of 10. As shown in Figure 2a, sodium lactate supported the highest cell growth across all N sources (NO_3_^−^-N, NO_2_^−^-N, and NH_4_^+^-N). The maximum NO_3_^−^-N removal efficiency (98.9%) was achieved using acetate or lactate as the carbon source, consistent with the performance of *Thauera* sp. SND5 under identical carbon conditions (Wang and He, 2020). Complete NO_2_^−^-N removal occurred with succinate or lactate, but the NO_2_^−^-N removal efficiency was only 24.3% when acetate served as the carbon source. The results indicated that JM12B12 exhibited distinct optimal carbon source preferences in denitrification processes aimed at removing NO_2_^−^-N as opposed to NO_3_^−^-N. This metabolic divergence was likely attributed to differential enzymatic activities and energy-yielding efficiencies associated with the two N sources during the dissimilatory nitrate reduction pathway (Lu et al., 2024). Unfortunately, JM12B12 exhibited relatively weak NH_4_^+^-N removal capacity, achieving a maximum removal efficiency of only 30.7% when lactate served as the carbon source. Notably, no inorganic N accumulation was observed when any single N source was removed. Based on these results, sodium lactate was selected as the optimal carbon source for subsequent experiments.

The C/N ratio significantly influenced bacterial growth, denitrification efficiency, and environmental adaptability (Gu et al., 2022). To determine the optimal ratio for JM12B12, we tested C/N ratios from 1 to 20 (Figure 2b). For NO_3_^−^-N removal, the efficiency improved from 15.3 to 100% when the ratio increased from 1 to 5. Complete NO_3_^−^-N removal occurred at ratios ≥8, with 99.5% efficiency maintained at a ratio of 20. No NO_2_^−^-N or NO_3_^−^-N accumulated during this process. For NO_2_^−^-N removal, the efficiency improved from 30.0 to 100% when the ratio increased from 1 to 5. And complete NO_2_^−^-N removal was consistently observed across C/N ratios of 8 to 20. No NO_3_^−^-N or NH₄⁺-N accumulation during this process. In general, the majority of aerobic denitrifiers exhibited high denitrification efficiency at C/N ratios of 5 or above, such as *P. balearica* strain RAD-17 (optimal ratio of 7.5), *P. denitrificans* XW11 (optimal ratio of 10), and *Pseudomonas* sp. Y15 (optimal ratio of 15) (Chen J. et al., 2024; Wu Y. et al., 2024; Zhang et al., 2023b). The required C/N ratio of JM12B12 was significantly lower than that of these strains, indicating that JM12B12 possessed efficient denitrification capability under low C/N ratio conditions. For NH_4_^+^-N removal, the efficiency rose slightly from 14.7 to 30.2% as the C/N ratio increased from 1 to 5, remaining stable (ranging from 29.0 to 36.3%) at ratios of 5 to 20. These results suggested that JM12B12 exhibited inefficient NH_4_^+^-N removal. It was suggested that this strain removed NH_4_^+^-N primarily through assimilation rather than via the heterotrophic nitrification-aerobic denitrification pathway, a finding consistent with the behavior observed in denitrifiers such as *Klebsiella* sp. TSH15, *P. denitrificans* R-1, and *P. mosselii* 9–1 (Cai et al., 2023; Ren J. et al., 2024; Sun et al., 2024).

#### 3.2.2 Effect of various environmental factors on denitrification

**Figure 3:**
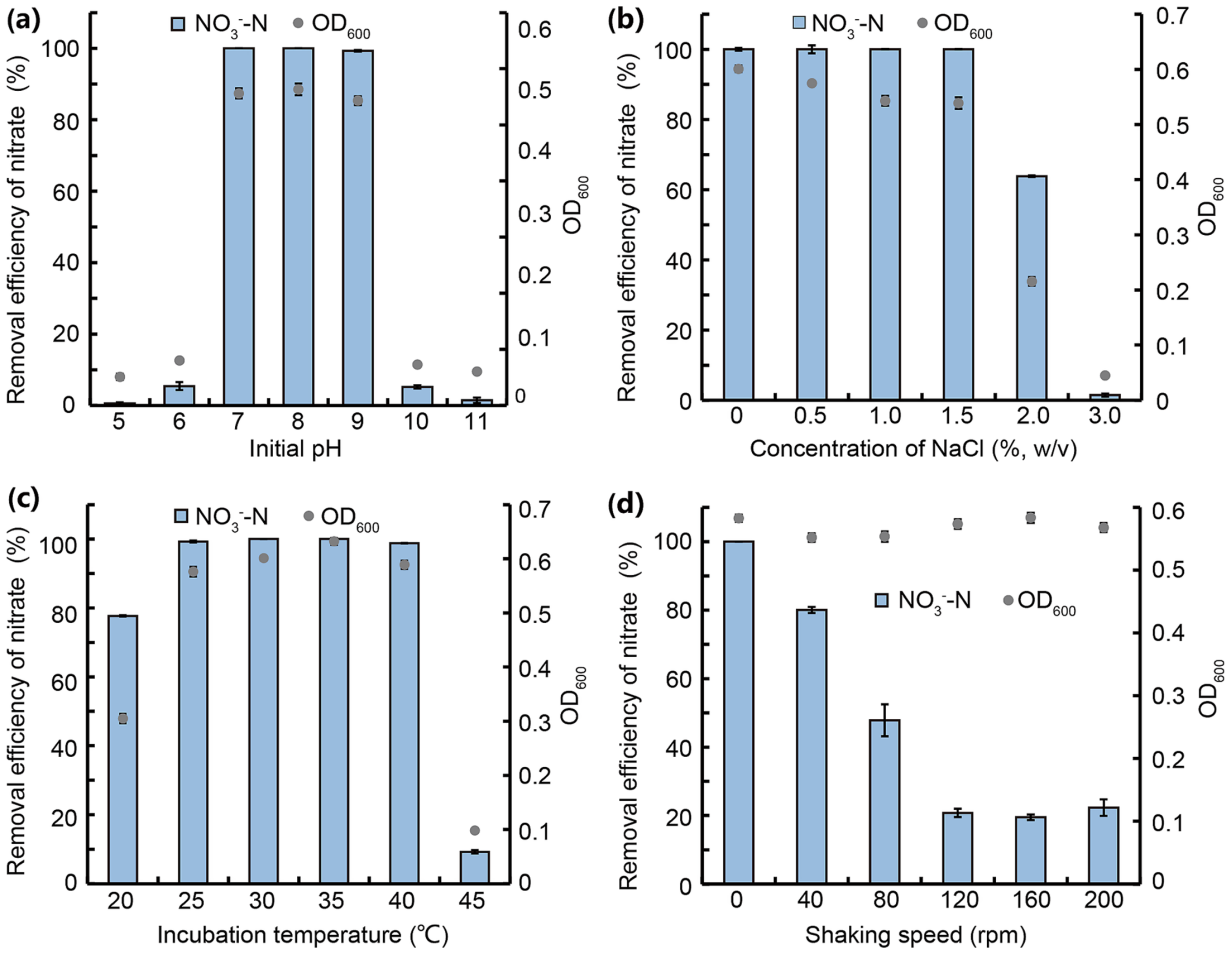
NO_3_^−^-N removal by strain JM12B12 in denitrification media across varied conditions. **(a)** Initial pH. **(b)** Concentration of NaCl. **(c)** Incubation temperature. **(d)** Shaking speed. Data shown as mean ± SD.

Initial pH critically influenced bacterial denitrification by regulating enzyme activity (Yue et al., 2023). As shown in Figure 3a, JM12B12 achieved near-complete NO_3_^−^-N removal (100, 100, 99.3%) at pH 7 to 9, but efficiency plummeted to 5.5 and 5.2% at pH 6 and 10, respectively, with negligible removal observed at pH 5 and 11. It was suggested that the slightly acidic and strongly alkaline environment negatively affected denitrification enzymic activities and caused the N metabolic imbalance of bacteria (Lan et al., 2023; Pan et al., 2023). JM12B12 exhibited optimal NO_3_^−^-N removal efficiency at pH 7–9 (neutral to slightly alkaline), consistent with *P. mendocina* A4 and *Pelomonas puraquae* WJ1 (Shu et al., 2024; Wu T. et al., 2024).

Salinity significantly influenced cellular osmotic pressure and microbial enzymatic activity (Hu et al., 2023). As shown in Figure 3b, JM12B12 exhibited complete NO_3_^−^-N removal (0–1.5% NaCl) without detectable NO_2_^−^-N or NH_4_^+^-N accumulation, a performance contrasting with other reported denitrifiers that exhibit metabolic inhibition under similar high-salinity (1.3%) conditions (Li et al., 2022). However, the NO_3_^−^-N removal efficiency was reduced to 63.8% when NaCl concentration was elevated to 2.0% (also without detectable NO_2_^−^-N or NH_4_^+^-N accumulation), and it was completely inhibited at 3.0%. For JM12B12, high salt might inhibit the activity of nitrate reductase rather than that of nitrite reductase. Based on the OD_600_ values, JM12B12 was unable to grow at 3.0% NaCl. Therefore, we hypothesized that high salt might lead to the death of JM12B12 by altering cellular osmotic pressure, ultimately resulting in its poor denitrification performance. These results indicated that JM12B12 might be tolerant of a certain salinity, but the high salinity (above 2.0%) had a significant and negative impact on the NO_3_^−^-N reduction capability of this strain. Based on farmers’ experiences and researchers’ findings, pond water for breeding shrimp should maintain proper salinity. For instance, Kumar et al. (2023) reported that shrimp cultured at a salinity of 15 g/L exhibited better growth, survival, and feed efficiency. Therefore, JM12B12 possessed promising potential for removing excessive N from the culture water of shrimp.

Temperature, as an important influencing factor on denitrification, delayed the regulation of denitrification key genes and inhibited enzymatic activity (Yang et al., 2020). As shown in Figure 3c, JM12B12 completely removed NO_3_^−^-N at 30 °C and 35 °C with no NO_2_^−^-N or NH_4_^+^-N accumulation. It also achieved 99.3 and 98.8% removal efficiencies at 25 °C and 40 °C, respectively. But the NO_3_^−^-N removal efficiencies dropped significantly to 77.7% at 20 °C and 9.2% at 45 °C. This phenomenon might be attributed to the suppression of denitrifying bacterial enzyme activities, cell proliferation, and metabolic processes under conditions of excessively low or high temperature (Liao et al., 2021). Our results indicated that JM12B12 adapted effectively to a certain temperature range (25–40 °C), consistent with the performance of most denitrifying bacteria (Wang et al., 2024; Yang et al., 2023).

To investigate the effect of DO concentration on the denitrification of JM12B12, the NO_3_^−^-N removal performance of this strain under varying shaking speed conditions was evaluated. As shown in Figure 3d, JM12B12 completely removed NO_3_^−^-N with no NO_2_^−^-N or NH_4_^+^-N accumulation at 0 rpm. At 40 and 80 rpm, removal efficiencies dropped to 80.0 and 47.8%, with trace NO_2_^−^-N accumulation. At 120–200 rpm, shaking speed had no further impact on NO_3_^−^-N removal, but efficiency fell to 20.9%, accompanied by slight NO_2_^−^-N accumulation (~6.6 mg/L). Our results demonstrated that the DO concentration influenced the denitrification process of JM12B12. However, this influence was less pronounced compared to that on traditional denitrifying bacteria, which are strictly anaerobic (Lou et al., 2023). Therefore, JM12B12 was a microaerobic denitrifying bacterium that exhibited high denitrification efficiency under low concentrations of DO, which was inconsistent with most aerobic denitrifiers, such as *P. versutus* JUST-3, *Stutzerimonas stutzeri* os3, and *Bacillus* sp. L2 (Li et al., 2024; Wang F. et al., 2025; Yan et al., 2025). Strain JM12B12 could potentially be utilized for N removal from the aquaculture wastewater in biofilters (without aeration devices) within RASs.

#### 3.2.3 Denitrification process and nitrogen balance

**Figure 4:**
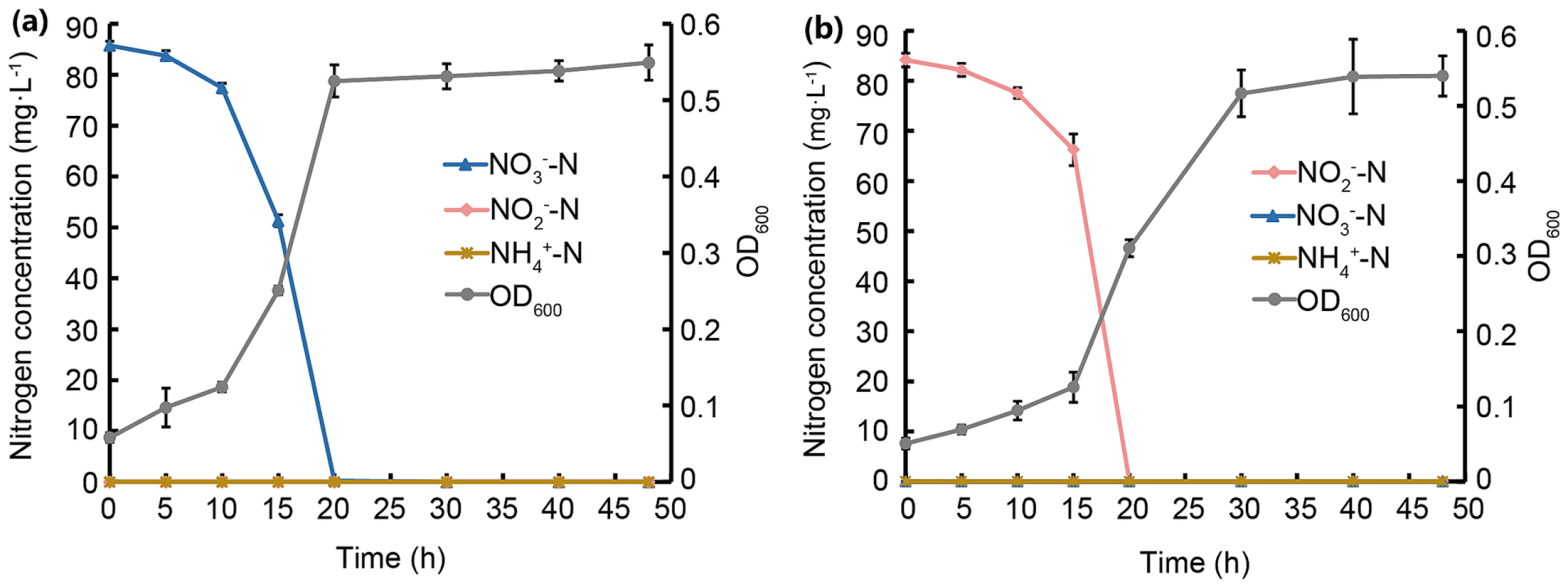
Processes of NO_3_^−^-N and NO_2_^−^-N removal and growth of strain JM12B12. **(a)** NO_3_^−^-N. **(b)** NO_2_^−^-N. Data shown as mean ± SD.

Growth and denitrification of JM12B12 were investigated JM12B12, showing a strong correlation between its growth rates and N substrate removal rates. As shown in Figure 4a, the OD_600_ values increased from 0.1 to 0.7, and the NO_2_^−^-N was completely removed after 20 h of incubation. Correspondingly, the average NO_2_^−^-N removal rate of this strain was determined to be 4.2 mg/L/h, exceeding that of *Halomonas* sp. DN3 (0.95 mg/L/h), *Peribacillus* sp. EM-C3 (2.2 mg/L/h), and *Glutamicibacter arilaitensis* EM-H8 (3.9 mg/L/h) (Chen et al., 2023; Liang et al., 2023; Xie et al., 2023), but lower than that of *A. johnsonii* EN-J1 (6.8 mg/L/h) (Zhang et al., 2023a). As shown in Table 1, when NO_2_^−^-N served as the sole N source, the TN-N concentration decreased from 84.0 mg/L to 5.2 mg/L, achieving a TN-N removal efficiency of 93.8% (ΔTN-N = 78.8 mg/L). Concurrently, the CN-N concentration increased from 2.9 mg/L to 12.8 mg/L (ΔCN-N = 9.9 mg/L), implying that 11.8% of the initial TN-N (9.9 mg/L of 84.0 mg/L) was assimilated by JM12B12 for biomass synthesis. 82.0% of the initial TN-N (68.8 mg/L) might be converted to gaseous N products (N_2_, N_2_O, NO), accounting for the observed loss from the culture medium. This suggested that the N removal process was dominated by the denitrification pathway, with a minor fraction diverted to microbial growth.

**Table 1:** Nitrogen balance of strain JM12B12 under different nitrogen source conditions.

Media		NO_2_^−^-N (mg/L)	NO_3_^−^-N (mg/L)	TN-N (mg/L)	CN-N (mg/L)	Gas-N (mg/L)
BM1	Initial	84.2 ± 1.4	0	84.0 ± 0.3	2.9 ± 0.3	-
	After 48 h	0	0	5.2 ± 0.9	12.8 ± 0.5	68.8 ± 0.5
BM2	Initial	0	85.7 ± 0.8	84.1 ± 1.2	3.0 ± 0.3	-
	After 48 h	0	0	5.8 ± 1.4	13.3 ± 0.9	68.4 ± 0.8

Data shown as mean ± SD (*n* = 4). TN-N, total nitrogen; CN-N, intracellular N. Gas-N, gaseous nitrogen. Gas-N = [(TN-N)^A^ - (TN-N)^B^] - [(CN-N)^B^ - (CN-N)^A^]. A and B represent the initial nitrogen concentration and the nitrogen concentration after 48 h of incubation, respectively.

As shown in Figure 4b, JM12B12 entered the stationary growth phase after 20 h of incubation (OD_600_ = 0.9), coinciding temporally with the complete removal of NO_3_^−^-N. The average NO_3_^−^-N removal rate was 4.3 mg/L/h during the first 20 h, which was similar to that of *G. arilaitensis* EM-H8, and significantly higher than that of *Halomonas* sp. DN3 (1.9 mg/L/h), *Pseudomonas* sp. G16 (2.9 mg/L/h), and *Stutzerimonas* sp. X87 (3.8 mg/L/h) (Gao et al., 2023; Huang et al., 2025; Xie et al., 2023). Notably, no significant NO_2_^−^-N or NH_4_^+^-N accumulation was observed throughout the N removal process, a distinguishing feature of JM12B12 that underscores its performance of no secondary pollution. This phenomenon differed from that observed in some aerobic denitrifiers, which typically accumulated NO_2_^−^-N or NH_4_^+^-N during NO_3_^−^-N removal process (Huang et al., 2023; Mao et al., 2025). As shown in Table 1, when NO_3_^−^-N served as the sole N source, the TN-N concentration decreased from 84.1 mg/L to 5.8 mg/L, achieving a TN-N removal efficiency of 93.1% (ΔTN-N = 78.3 mg/L). The CN-N concentration increased from 3.0 mg/L to 13.3 mg/L (ΔCN-N = 10.3 mg/L), implying that 12.2% of the initial TN-N was assimilated by JM12B12 for biomass synthesis. 80.9% of the initial TN-N (68.4 mg/L) might be converted to gaseous N products, accounting for the observed loss from the culture medium.

Overall, *Thauera* sp. JM12B12, a micro-aerobic denitrifying bacterium, demonstrated higher removal efficiencies for NO_2_^−^-N, NO_3_^−^-N, and TN-N during denitrification compared to other denitrifying bacteria, such as *P. puraquae* WJ1, *A. johnsonii* EN-J1, and *Pseudomonas* sp. B-1 (Lan et al., 2023; Wu T. et al., 2024; Zhang et al., 2023a). Excess NO_3_^−^-N was prevalent in the intensive RASs, necessitating cost-effective and eco-friendly wastewater treatment for sustainable aquaculture. Therefore, JM12B12 exhibited significant potential as a denitrifying bacterium for the treatment of aquaculture effluents, especially within RASs.

### 3.3 Characterization of genes in the denitrification pathway of JM12B12

**Figure 5:**
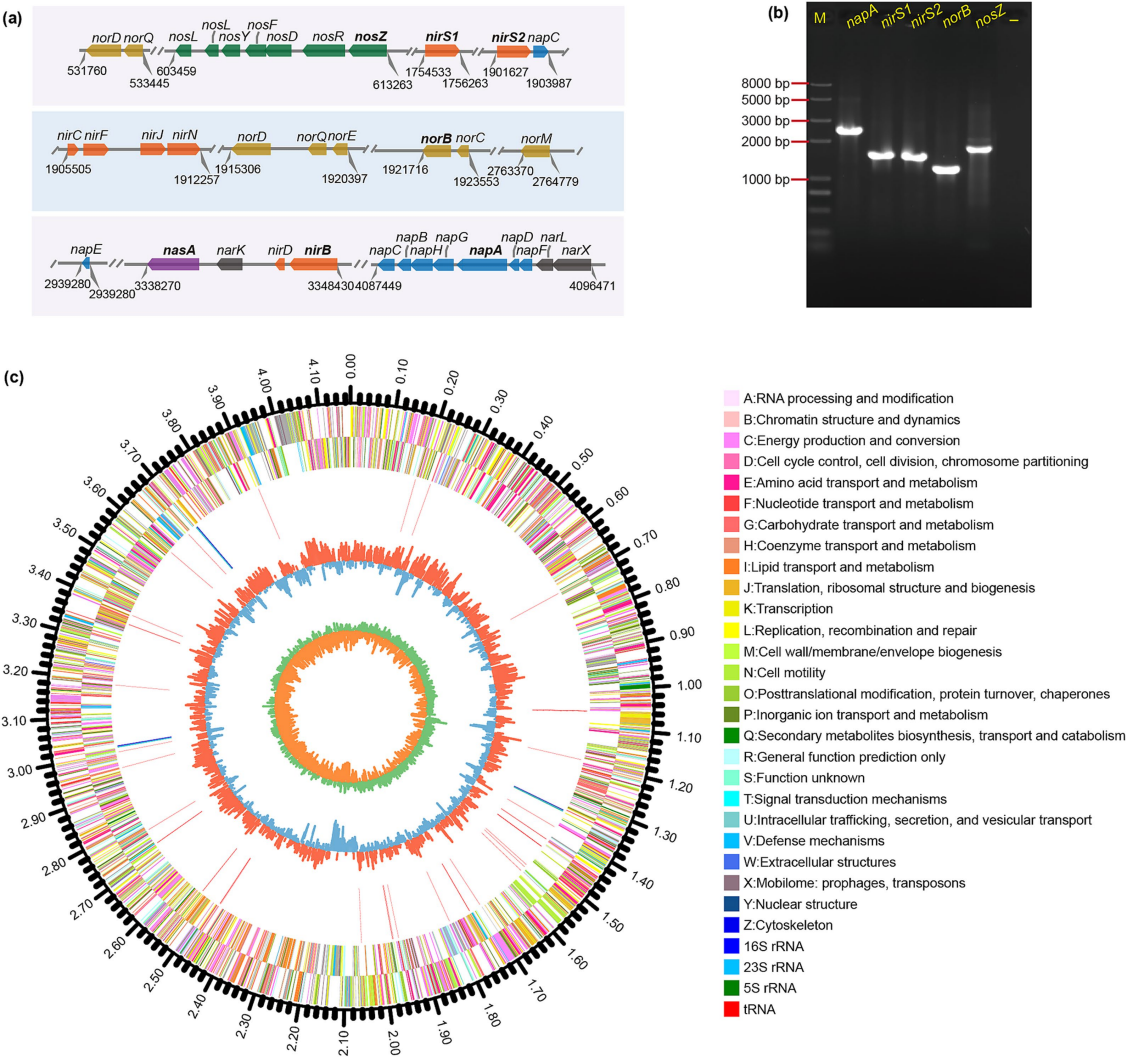
Genomic circular map and functional genes associated with aerobic denitrification in strain JM12B12. **(a)** Denitrification gene layout, with parallel lines marking sequence breaks and numbers indicating loci. **(b)** PCR results for aerobic denitrification key genes. -, negative control. **(c)** Circos plot of the closed circular genome, featuring concentric layers: genome size scale, CDS on both strands (color-coded by COG categories), rRNA/tRNA positions, GC content, and GC skew.

Denitrifying bacteria require an array of reductases to accomplish the denitrification pathway. According to RAST annotation of genome, 36 genes were associated with denitrification and nitrite/nitrate assimilation pathways (Figure 5a). Dissimilatory nitrate reduction to nitrite was catalyzed by two types of enzymes: the membrane-bound nitrate reductase (Nar) and the periplasmic nitrate reductase (Nap), and Nar and Nap reductases were often associated with anaerobic and aerobic denitrification, respectively (Hu et al., 2023). A *napFDAGHBC* gene cluster (gene_3698/3697/3696/3695/3694/3693/3692) encoding NapA and relevant enzymes was identified in JM12B12, which was consistent with *Shewanella oneidensis* MR-1 (Liu et al., 2021). Dissimilatory nitrite reduction to nitric oxide was catalyzed by two structurally distinct enzymes: a cytochrome *cd1*-dependent nitrite reductase (encoded by *nirS*) and a copper-containing nitrite reductase (encoded by *nirK*). Interestingly, two *nirS* gene (gene_1573 and gene_1693) sequences with a similarity of 66.2% were found in the genome of JM12B12, which was inconsistent with *Bradyrhizobium diazoefficiens* (Pacheco et al., 2022). Previous studies have demonstrated that *nirS*-type denitrifying bacteria possessed robust metabolic systems for energy conservation, facilitating their survival under environmental stresses (Ming et al., 2024). The genome of JM12B12 harbored a functionally enriched cluster of energy metabolism-associated genes, demonstrating evolutionary adaptations critical for environmental persistence. The key functional genes for reducing NO to nitrous oxide (N_2_O) (*norB*, gene_1714) and reducing N_2_O to N_2_ (*nosZ*, gene_0523) were also identified.

Furthermore, the key genes involved in denitrification were amplified via PCR from the genomic DNA of JM12B12. As shown in Figure 5b, the *napA*, *nirS1*, *nirS2*, *norB*, and *nosZ* genes were determined via agarose gel electrophoresis. The results were consistent with the predicted sizes of the relevant functional genes within the JM12B12 genome assembly. Overall, the strain JM12B12 harbored all the denitrification genes, which indicated its potential for complete N removal through the denitrification pathway: NO_3_^−^-N → NO_2_^−^-N → NO→N_2_O → N_2_.

### 3.4 Flocculation characterization of JM12B12

**Figure 6:**
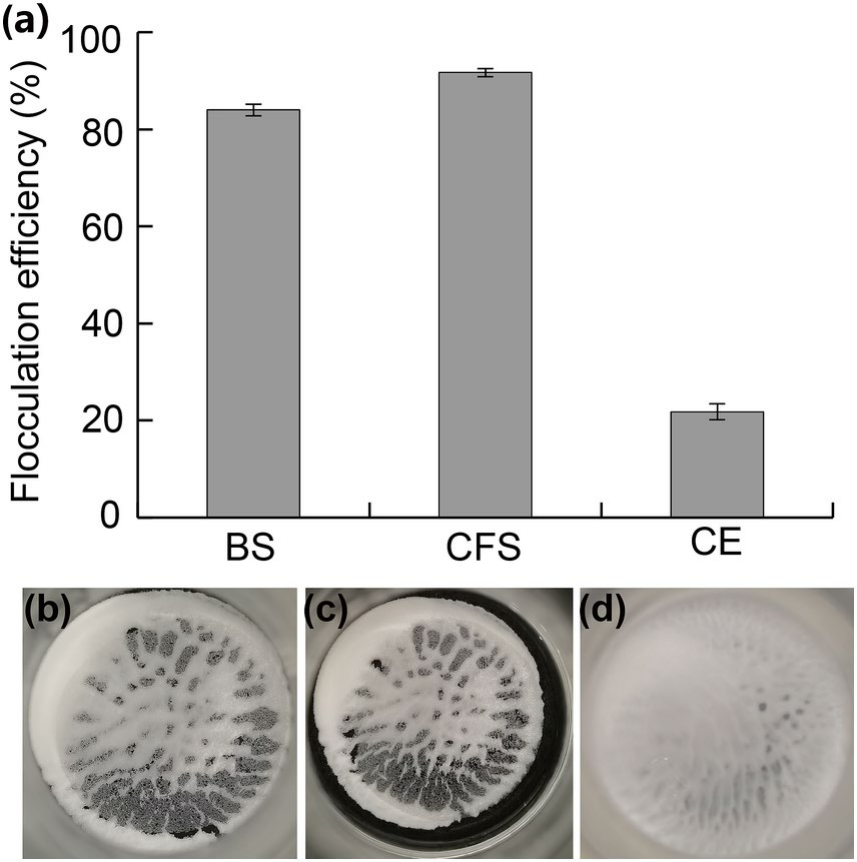
Flocculating activity of bacterial suspension (BS), cell-free supernatant (CFS), and cells (CE) of strain JM12B12. **(a)** Flocculation efficiencies. **(b–d)** Images of kaolin flocs with BS, CFS, and CE, respectively. Data shown as mean ± SD.

Bioflocculants produced by different bacteria were various in their distribution. Some were secreted into the extracellular environment, and others were tightly adhered to the cellular surface. To investigate the flocculation characterization of JM12B12, the BS, CFS, and CE were obtained after it grew in the denitrification medium. As shown in Figure 6, the flocculation efficiencies of BS, CFS, and CE were 83.7, 91.4, and 21.8%, respectively. The results indicated that the flocculants produced by JM12B12 were found mainly in the cell-free supernatant, consistent with that of strains *Stenotrophomonas pavanii* GXUN74707, *Pseudomonas* sp. XD-3, and *Providencia huaxiensis* OR794369.1 (Chen L. et al., 2024; Qin et al., 2024; Selepe and Maliehe, 2024). Bioflocculants produced by bacteria were found to be EPS, which were mainly composed of polysaccharides, protein polymers, glycoproteins, etc. (Selepe and Maliehe, 2024). Therefore, JM12B12 was a novel denitrifying bacterium capable of producing bioflocculants, suggesting that it possesses important features in practical applications of bioremediation and wastewater treatment.

### 3.5 Profiling of potential flocculation genes based on the genome of JM12B12

Our results indicated that JM12B12 was capable of producing extracellular bioflocculants. In general, bacterial bioflocculants were composed of EPS, which included polysaccharides, proteins, extracellular nucleic acids, and lipids, and extracellular polysaccharides (exopolysaccharides) constituted the dominant component of EPS (Flemming et al., 2025; Vandana and Das, 2023). Therefore, the genes associated with exopolysaccharide biosynthesis and protein secretion systems were identified by analyzing the genome annotations of JM12B12. Exopolysaccharides production was a multistage process where polysaccharides were intracellularly synthesized and exported outside (Stephens et al., 2023). According to analyses of annotations, there were 31 genes related to polysaccharide biosynthetic, polysaccharide export, and biopolymer transport processes, such as genes *alg*, *rfb*, *udg*, *wbp*, *eps*, *exb*, and *tol*, etc. (Supplementary Table S3). These genes might be essential for exopolysaccharide production of JM12B12. Extracellular proteins, one of the key amphiphilic macromolecules within EPS, played a crucial role in governing flocculating ability (He et al., 2024). Secretion systems were protein export machines that enable bacteria to exploit their environment through the release of protein effectors (Lauber et al., 2024). There were 49 genes identified as components of the bacterial protein secretion machineries, encompassing the Sec (general secretory pathway) and Tat (twin-arginine translocation) systems, along with Type I, II, III, IV, and VI secretion systems (Supplementary Table S4). Overall, the genes involved in exopolysaccharides production and protein secretion systems might be the potential functional genes associated with the flocculating activity of JM12B12. However, the mechanism of bioflocculant synthesis in this bacterium remains to be fully clarified in the future.

### 3.6 Comparative genomic analysis and metabolic reconstruction of JM12B12

The circus map of genome characteristics of JM12B12 was comprehensively displayed in Figure 5c. The genomic analysis of JM12B12 revealed a single circular chromosome spanning 4,171,389 bp, characterized by a GC content of 67.9%. Notably, no plasmid was identified in this bacterium. The absence of plasmid reduced the probability of genes (such as genes related to antimicrobial resistance) transfer within strain JM12B12, potentially resulting in enhanced safety compared to the plasmid-harboring bacteria. Genome data has been deposited in the NCBI GenBank database under the accession number CP154859. Compared with related strains in the genus *Thauera*, JM12B12 had ANI values of 84.2–87.8% and dDDH values of 26.3–31.4% (Figures 1d,e), which were all below the classical standard species delineation threshold values of 95.0 and 70%, respectively (Goris et al., 2007; Richter and Rosselló-Móra, 2009). The phylogenomic tree constructed using UBCGs indicated that JM12B12 fell in a large clade with members of the genus *Thauera*, which was consistent with the phylogenetic relationships inferred from 16S rRNA gene sequence analysis (Supplementary Figure S2). Moreover, JM12B12 together with *T. aminoaromatica* S2 and *T. phenylacetica* B4P formed a clade supported by the bootstrap value of 100%. Phylogenomic analyses incorporating dDDH values, ANI values, and phylogenetic reconstruction conclusively confirmed that strain JM12B12 was a novel species in the genus *Thauera* (Riesco and Trujillo, 2024).

Aggregate annotation results indicated that a total of 3,771 genes, including 3,707 protein-coding sequences (CDS), 55 tRNA genes, and 9 rRNA genes, were identified. The metabolic pathways were analyzed based on genome annotations. The RAST annotation showed that there were 363, 301, 300, and 277 genes associated with ‘Amino acids and derivatives’, ‘Cofactors, vitamins, prosthetic groups, pigments’, and ‘Carbohydrates’, respectively (Supplementary Figure S3). The KEGG annotation showed that the top four pathways with the most enriched number of genes were ‘Global and overview maps’, ‘Energy metabolism’, and ‘Amino acid metabolism’, respectively (Supplementary Figure S4). The COG annotation revealed that 287, 282, 282, and 259 genes were categorized under the functional classes ‘Energy production and conversion’, ‘Amino acid transport and metabolism’, and ‘Signal transduction mechanisms’, respectively (Supplementary Figure S5). The reconstruction of this bacterium’s carbon metabolic pathways based on genome annotation revealed that the tricarboxylic acid cycle (TCA), glyoxylate cycle (GAC), glycolysis pathway (Embden-Meyerhof-Parnas, EMP), and gluconeogenesis pathway (GNG) were all complete. These carbon metabolic pathways could supply electrons for denitrification and provide precursors for the synthesis of extracellular polysaccharides and proteins.

Our research studied *Thauera* sp. JM12B12, an aerobic denitrifying bacterium that uniquely combines the capability to produce bioflocculants with efficient denitrification, even under low C/N ratio conditions. Consequently, we proposed that JM12B12 held significant promise as a candidate for wastewater treatment in RASs. However, it is important to acknowledge the limitations of our study, which can be addressed and supplemented in the future. Firstly, we will elucidate the chemical composition and mechanisms of EPS produced by JM12B12. To advance the practical application of *Thauera* sp. JM12B12 in N removal from aquaculture water, future research could focus on assessing its biosafety, validating its denitrification efficiency in real RASs aquaculture water. Additionally, employ the molecular biology, genetic biology, transcriptomics, and metabolomics techniques to elucidate the underlying mechanisms behind JM12B12’s flocculation and its exceptional denitrification performance at low C/N ratios.

## 4 Conclusion

*Thauera* sp. JM12B12, a novel denitrifying bacterium with bioflocculant-producing capability, demonstrated high NO_3_^−^-N and NO_2_^−^-N removal and bioflocculation efficiency under low C/N ratio conditions. Using sodium lactate as the sole carbon source, it achieved complete removal of NO_2_^−^-N and NO_3_^−^-N at a low C/N ratio of 5, with TN-N removal efficiencies reaching 93.8 and 93.1%, respectively. Optimal denitrification occurred across a broad pH range (7–9), salinity (0–1.5%), and temperature (25–40 °C) under static conditions. Additionally, the cell-free supernatant during NO_3_^−^-N removal exhibited a high flocculation activity of 91.4%. Genome analyses revealed a complete denitrification pathway and 80 potential functional genes associated with the production of bioflocculants, highlighting its dual functionalities for removing N and suspended particles from wastewater.

## Data Availability

The datasets presented in this study can be found in online repositories. The names of the repository/repositories and accession number(s) can be found in the article/Supplementary material.
