# Supplementary material for: Dual-functionality of Thauera sp. JM12B12: aerobic denitrification and bioflocculation for nitrogen and suspended particles removal at low carbon-to-nitrogen ratios
Source: Front Microbiol. 2025 Dec 18;16:1730924. doi: 10.3389/fmicb.2025.1730924 (PMC12756365; doi:10.3389/fmicb.2025.1730924)
Supplement: Supplementary file 1 [file Data_Sheet_1.pdf]

Supplemental Information

Dual-functionality of *Thauera* sp. JM12B12: Aerobic denitrification and bioflocculation for nitrogen and suspended particles removal at low carbon-to-nitrogen ratios.

Mingxia Zhang[1], Yulian Zhang[1], Qing Yao[2], Yanna Hu[1], Honghui Zhu[1,*]

[1] Key Laboratory of Agricultural Microbiomics and Precision Application (MARA), Key Laboratory of Agricultural Microbiome (MARA), Guangdong Microbial Culture Collection Center (GDMCC), Guangdong Provincial Key Laboratory of Microbial Culture Collection and Application, State Key Laboratory of Applied Microbiology Southern China, Institute of Microbiology, Guangdong Academy of Sciences, Guangzhou 510070, P. R. China.

[2] Key Laboratory of Biology and Genetic Improvement of Horticultural Crops (South China), Ministry of Agriculture and Rural Affairs, Guangdong Province Key Laboratory of Microbial Signals and Disease Control, Guangdong Engineering Research Center for Litchi, College of Horticulture, South China Agricultural University, Guangzhou, 510642, P. R. China.

* Corresponding author (H. Zhu). *E-mail address*: zhuhh_gdim@163.com.

Table S1. Bacteria isolated from the aquaculture water for *Litopenaeus vannamei* and their NO_2^--N removal capabilities.

**Strains**	**Top-hit type strains**	**Similarity (%)**	**NO_2^--N removal capabilities**
JM12B1	*Acinetobacter venetianus* RAG-1^T	99.6	–
JM12B10	*Aeromonas veronii* CECT 4257^T	100	w
JM12B3	*Cellvibrio mixtus* ACM 2601^T	98.8	–
JM12B11	*Hydrogenophaga bisanensis* K102^T	99.1	–
JM12B9	*Pseudaeromonas paramecii* PCS8^T	99.5	–
JM12B4b	*Pseudoclavibacter triregionum* 125703-2019^T	99.6	–
JM12B2	*Pseudomonas tianjinensis* 68^T	99.1	–
JM12B4a	*Pseudomonas xionganensis* R-22-3w-18^T	100	w
JM12B7	*Pseudomonas xionganensis* R-22-3w-18^T	99.9	w
JM12B12	*Thauera chlorobenzoica* 3CB-1^T	99.1	+

Note: Qualitative determination of NO_2^--N via Griess reaction. –, appeared purple-red. w, appeared pink. +, colourless.

Table S2. List of primers used in this study.

Gene name (ID)	Primers name	Sequence (5'→3')	Length (bp)
napA (gene_3696)	napA_5F	CATCAAGAACAACGCGATTG	2547
	napA_3R	GGCCTTGACCACCTTCAC	
nirS1 (gene_1573)	nirS1_5F	GGTGACGAGGCGCTTAAG	1731
	nirS1_3R	GTTGTGGACGTTGAACTTGC	
nirS2 (gene_1693)	nirS2_5F	GGTTCAACTCTGGCGATG	1683
	nirS2_3R	GAGGGTCTTGTCGTCGTAG	
norB (gene_1714)	norB_5F	CTGATCATGGGCCTGCAG	1380
	norB_3R	GAAGCTTGCGAGGTAGGTC	
nosZ (gene_0523)	nosZ_5F	CTTCAATACCGCGGCGCTG	1950
	nosZ_3R	CAGTAGCACCAGAACACCC	

Table S3. The functional genes associated with exopolysaccharide synthesis and excretion in the genome of strain JM12B12.

Gene ID	Gene name	COG Description
gene_1839	*algA*	Mannose-6-phosphate isomerase
gene_1009	*algD*	UDP-glucose 6-dehydrogenase
gene_3380	*algH*	Putative transcriptional regulator
gene_1029	*algI*	D-alanyl-lipoteichoic acid acyltransferase DltB
gene_1074	*algR*	DNA-binding response regulator
gene_1073	*algZ*	Sensor histidine kinase
gene_2951	*rfbA*	dTDP-glucose pyrophosphorylase
gene_1837	*rfbB*	dTDP-D-glucose 4,6-dehydratase
gene_2950	*rfbC*	dTDP-4-dehydrorhamnose 3,5-epimerase
gene_1838	*rfbD*	dTDP-4-dehydrorhamnose reductase
gene_1023	*rfbE*	dTDP-4-amino-4,6-dideoxygalactose transaminase
gene_1028	*ugd*	UDP-glucose 6-dehydrogenase
gene_3468	*wbpE*	dTDP-4-amino-4,6-dideoxygalactose transaminase
gene_3469	*wbpI*	UDP-N-acetylglucosamine 2-epimerase
gene_2709	*wbpO*	UDP-N-acetyl-D-mannosaminuronate dehydrogenase
gene_3464	*wbpO*	UDP-N-acetyl-D-mannosaminuronate dehydrogenase
gene_0798	–	Glycosyltransferase A (GT-A) superfamily
gene_3461	*wzc*	Exopolysaccharide export protein/domain GumC/Wzc1
gene_0994	*epsF*	Exopolysaccharide export protein/domain GumC/Wzc1
gene_3462	*wzzB*	Exopolysaccharide export protein/domain GumC/Wzc1
gene_0767	*exoD*	Exopolysaccharide synthesis protein ExoD
gene_0993	*gfcE*	Periplasmic protein Wza involved in polysaccharide export
gene_3470	–	ABC-type polysaccharide export permease
gene_0847	*exbB*	Biopolymer transport protein ExbB/TolQ
gene_2733	*exbB*	Biopolymer transport protein ExbB/TolQ
gene_0846	*exbD*	Biopolymer transport protein ExbD
gene_2732	*exbD*	Biopolymer transport protein ExbD
gene_0632	*tolB*	Periplasmic component TolB of the Tol biopolymer transport system
gene_0089	*tolB*	Periplasmic component TolB of the Tol biopolymer transport system
gene_0635	*tolQ*	Biopolymer transport protein ExbB/TolQ
gene_0634	*tolR*	Biopolymer transport protein ExbD

Table S4. The functional genes associated with protein secretion in the genome of strain JM12B12.

Gene ID	Gene name	COG Description
gene_3071	*secA*	Preprotein translocase subunit SecA (ATPase, RNA helicase)
gene_1140	*secB*	Preprotein translocase subunit SecB
gene_3065	*secD*	Preprotein translocase subunit SecD
gene_0927	*secE*	Preprotein translocase subunit SecE
gene_3066	*secF*	Preprotein translocase subunit SecF
gene_1520	*secG*	Protein translocase subunit SecG
gene_0960	*secY*	Preprotein translocase subunit SecY
gene_3212	*tatA*	Twin-arginine protein secretion pathway components TatA and TatB
gene_3211	*tatB*	Twin-arginine protein secretion pathway components TatA and TatB
gene_3210	*tatC*	Twin-arginine protein secretion pathway component TatC
gene_1296	*tolC*	Outer membrane protein TolC/type I secretion system protein
gene_3110	*tolC*	Outer membrane protein TolC/type I secretion system protein
gene_0877	*lapB*	ABC-type bacteriocin/lantibiotic exporters/type I secretion system permease
gene_0878	*lapC*	Multidrug resistance efflux pump EmrA/type I secretion periplasmic adaptor subunit
gene_0997	*gspA*	Type II secretory pathway ATPase component GspA/ExeA/MshM
gene_0643	*gspE*	Type II secretory pathway ATPase GspE/PulE
gene_2982	*pilB*	Type II secretory pathway ATPase GspE/PulE
gene_3201	*pilB*	Type II secretory pathway ATPase GspE/PulE
gene_3235	*pilB*	Type II secretory pathway ATPase GspE/PulE
gene_3234	*mshL*	Type II secretory pathway component GspD/PulD (secretin)
gene_0648	*gspD*	Type II secretory pathway, component HofQ
gene_0706	*pilQ*	Type II secretory pathway, component HofQ
gene_0642	*gspF*	Type II secretory pathway, component PulF
gene_2983	*pilC*	Type II secretory pathway, component PulF
gene_3236	*pilC*	Type II secretory pathway, component PulF
gene_0641	*gspG*	Type II secretory pathway, pseudopilin PulG
gene_0649	*gspG*	Type II secretory pathway, pseudopilin PulG
gene_0650	*gspG*	Type II secretory pathway, pseudopilin PulG
gene_0651	*pulG*	Type II secretory pathway, pseudopilin PulG
gene_3239	*pulG*	Type II secretory pathway, pseudopilin PulG
gene_3240	*pulG*	Type II secretory pathway, pseudopilin PulG
gene_1621	*tadC*	Flp pilus assembly protein TadB/type II secretion system F family protein
gene_1622	*tadB*	Flp pilus assembly protein TadB/type II secretion system F family protein

gene_2984	*pilD*	Prepilin signal peptidase PulO (type II secretory pathway)
gene_3194	*tamB*	Autotransporter translocation and assembly protein TamB
gene_1385	*flhA*	Flagellar biosynthesis protein FlhA/type III secretion system protein FlhA
gene_1384	*flhB*	Flagellar biosynthesis protein FlhB/type III secretion system protein FlhB
gene_1428	*flhB2*	Type III secretion system substrate exporter, FlhB-like
gene_1412	*fliP*	Flagellar biosynthesis protein FliP/type III secretion system pore protein FliP
gene_1411	*fliQ*	Flagellar biosynthesis protein FliQ
gene_1419	*fliI*	Flagellar biosynthesis/type III secretory pathway ATPase FliI
gene_1395	*flgN*	Flagellar biosynthesis/type III secretory pathway chaperone FlgN
gene_1422	*fliF*	Flagellar biosynthesis/type III secretory pathway M-ring protein FliF/YscJ
gene_1420	*fliH*	Flagellar biosynthesis/type III secretory pathway protein FliH
gene_1414	*fliN*	Flagellar motor switch/type III secretory pathway protein FliN
gene_1431	*fliS*	Flagellin-specific chaperone FliS
gene_1624	*cpaC*	Flp pilus assembly protein, secretin CpaC
gene_3569	-	Type IV secretory pathway, VirD4 component, TraG/TraD family ATPase
gene_3618	*vgrG*	Conserved protein VgrG, implicated in type VI secretion and phage assembly

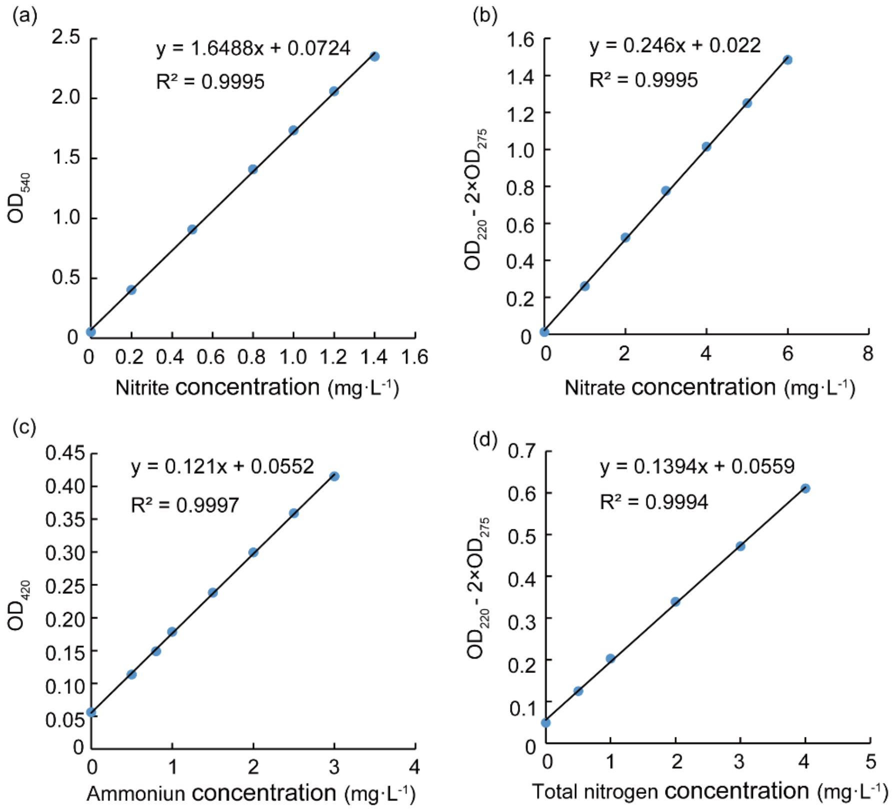

Figure S1. Standard curves for the determination of different nitrogen concentrations. (a), NO_2^--N. (b), NO_3^--N. (c), NH_4^+-N. (d), Total nitrogen.

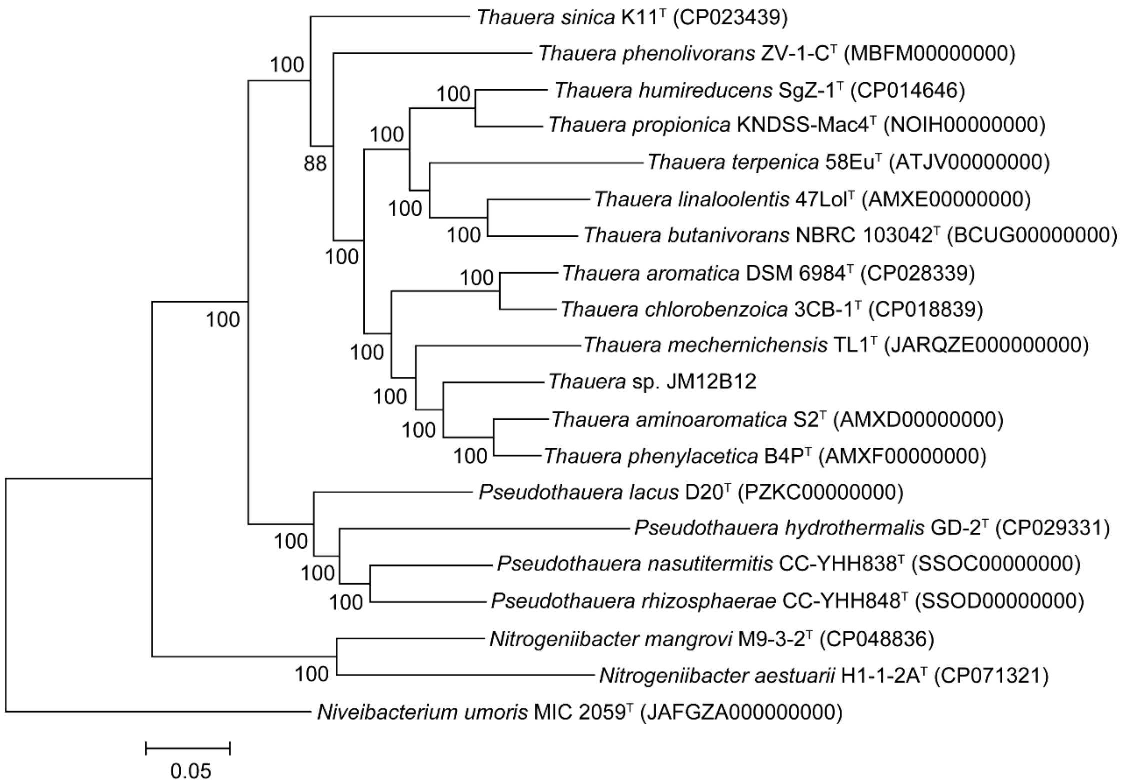

Figure S2. The phylogenomic tree based on the 92 bacterial core gene sets of strain JM12B12 and the related type strains. Type strain *Niveibacterium umoris* MIC 2059 was used as an outgroup. Bootstrap values higher than 70% were shown at branch points. Bar, 0.05 represents the number of substations per site.

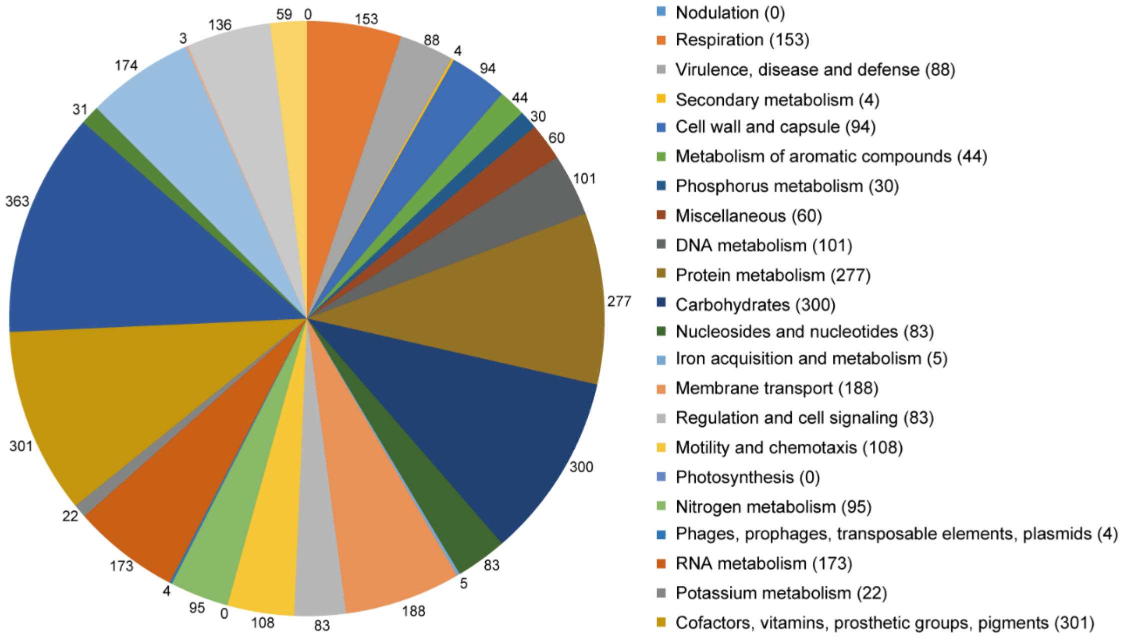

Figure S3. RAST annotation of strain JM12B12. The pie depicts the subsystem category distribution, and the numbers beside pie indicate the subsystem feature counts.

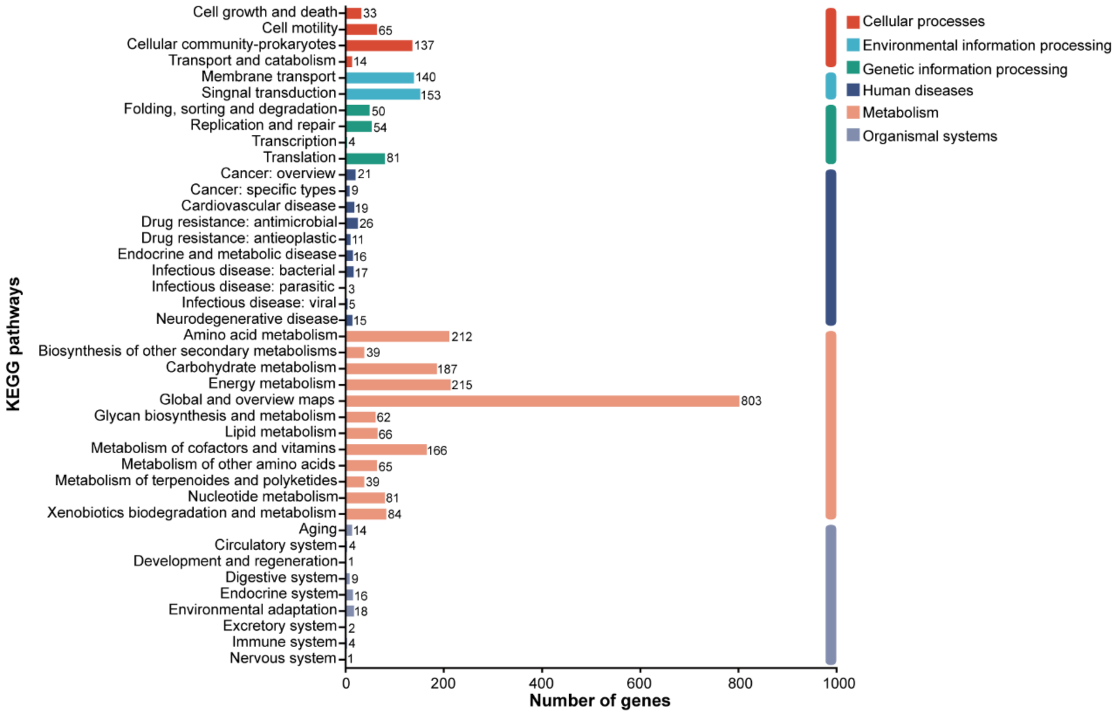

Figure S4. KEGG annotation of strain JM12B12. The vertical position indicates the level 2 classification of the KEGG pathway, and the horizontal axis represents the number of genes annotated under that classification. Different colors represent the level 1 classification of the KEGG pathway, and the sidebar numbers indicate the number of genes under different level 1 classifications.

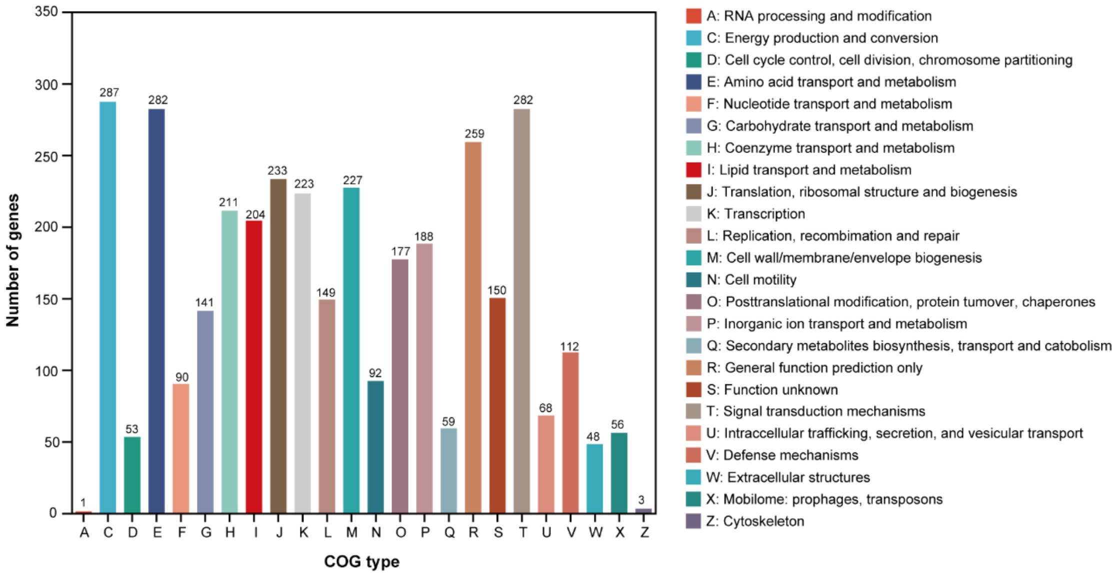

Figure S5. COG annotation of strain JM12B12. The legend on the right indicates detailed functional descriptions of each COG category. The sidebar numbers indicate the number of genes.
